# The healthy context paradox: a cross-country analysis of the association between bullying victimisation and adolescent mental health

**DOI:** 10.1007/s00787-024-02483-x

**Published:** 2024-06-04

**Authors:** Tracy Odigie, Esme Elsden, Mariko Hosozawa, Praveetha Patalay, Jean-Baptiste Pingault

**Affiliations:** Department of Clinical, Educational and Health Psychology, University College London, 26 Bedford Way, London, WC1H 0AP UK

**Keywords:** Bullying victimisation, Adolescent mental health, Cross-country analysis, Multi-level modelling, Healthy context paradox

## Abstract

Bullying victimisation is an increasing global health problem among adolescents and is associated with short- and long-term adverse mental health outcomes. Investigating whether associations with mental health vary across national contexts and why, can provide insights into mechanisms underlying those associations and inform policy. We used data from 479,685 adolescents participating in the 2018 Program for International Student Assessment (PISA) cross-sectional survey and examined whether the associations between bullying victimisation, psychological distress and life satisfaction vary across 63 countries. We further tested the modifying role of country-level factors – bullying prevalence, income inequality and national wealth, by implementing multilevel cross-country analyses. We found significant associations between bullying victimisation, increased psychological distress (β = 0.181; 95%CI: 0.178, 0.184) and decreased life satisfaction (β = -0.158; 95%CI: -0.162, -0.155). Associations between bullying victimisation, psychological distress and life satisfaction among adolescents were consistent across countries in terms of direction but effect sizes varied substantially. The effects ranged from β = 0.08 in the Philippines to β = 0.40 in South Korea for psychological distress and from β = −0.05 in the Philippines to β = −0.36 in the United Kingdom for life satisfaction. In addition, consistent with the “healthy context paradox” effect, associations between bullying and mental health were larger in countries where the prevalence of bullying was lower, as well as in higher-income countries. Interventions aiming to reduce bullying victimisation should aim to provide additional targeted support for those who still experience bullying after the intervention.

**Supplementary Information:**

The online version contains supplementary material available at 10.1007/s00787-024-02483-x.

## Introduction

Bullying victimisation is a large-scale public health concern amongst adolescents [1]. Defined as ‘exposure to repeated negative actions over time with the intent to cause harm’ [2], bullying victimisation refers to an asymmetric power relationship. Bullying can be categorised into three main subtypes: physical (e.g., hitting and damaging property), verbal (e.g., insults and teasing) and relational (e.g., spreading rumours and social exclusion) [3]. Bullying victimisation can occur in any social setting but it is commonly studied within school environments [4, 5], arguably the most important environment for socialisation outside the family for adolescents [6, 7]. Bullying victimisation is associated with short- and long-term adverse mental health outcomes [1, 8, 9], such as physical and psychological symptoms [10], poor emotional health [11], and psychosocial adjustment including loneliness and social anxiety [12].

Associations between bullying victimisation and mental health can be context dependent, as shown in particular by recent research on the healthy context paradox [13]. The healthy context paradox framework describes a phenomenon where living in a healthy context can exacerbate the adverse consequences of a given exposure for those still exposed. For example, in a context where bullying victimisation is rare, the few individuals who still experience bullying may be at heightened risk of developing adverse mental health outcomes [13]. Bellmore et al. found that in classrooms with low social disorder (classroom level of disruption, aggression and victimisation), the association between victimisation and anxiety was stronger [14]. Another study reported that victimised children experienced higher levels of somatic problems when in classrooms with lower levels of bullying victimisation [15]. A longitudinal analysis compared depression and anxiety levels of bullied victims across two time points, where in one classroom the proportion increased or remained the same across the two time points and in the other classroom the proportion decreased. Results highlighted that victimised adolescents in the classroom where the proportion of children who were bullied decreased felt more anxious and depressed than their counterparts [16]. These studies point towards this healthy context paradox where victims have greater adjustment problems in contexts where there is less victimisation overall.

Existing bullying victimisation studies regarding the health context paradox suffer from two limitations: (i) most focus on the micro context, mostly schools; (ii) the ‘healthy context’ is defined only by bullying levels. Here, we addressed the first limitation by undertaking cross-country analyses. In particular, we checked whether the country-level prevalence of bullying victimisation moderated the association between experiencing bullying and mental health outcomes, similarly to effects observed at the micro level. Cross-country analyses allow the examination of social determinants across countries, pointing to possible explanations for similarities and differences [17]. Such examination can provide key insights to policymakers, educators and clinicians that can be used to reduce bullying victimisation or its impact on mental health. We addressed the second limitation by not only focusing on bullying prevalence but by also examining other characteristics, typically associated with a healthy context, that may moderate the associations between bullying victimisation and mental health. We focused in particular on country-level income inequality and wealth.

Adolescents are at higher risks of being bullied in countries where income inequalities are larger [18]. Additionally, adolescents who reside in more economically unequal countries are at greater risk for poor mental health conditions such as depression and lower life satisfaction [19, 20]. Thus, there is evidence that income inequality is associated with experiencing bullying victimisation and poor mental health. Similarly, there is evidence that country-level income affects both the occurrence of bullying and the occurrence of mental health outcomes.

In summary, the present study examined the association between bullying victimisation and mental health and the moderating effect of bullying prevalence, income inequality and national wealth. Firstly, we examined associations between bullying victimisation, psychological distress and life satisfaction in all countries by examining the fixed effects of bullying victimisation across country, and the extent to which the random effects varied across countries. Secondly, we tested whether country-level factors explained some of the cross-country variation (i.e., whether such country-level factors acted as effect-modifiers, moderating these associations). We used data from the Programme for International Student Assessment (PISA) 2018 survey, a multi-country survey, using data from 63 countries. PISA 2018 used representative sampling where only those adolescents who were not in school at the age of 15–16 were excluded. By formally testing the moderating role of factors that have previously been associated with both bullying victimisation and mental health outcomes, we aimed to better understand if and why the association between bullying victimisation and adolescents’ mental health varied across the world.

## Methods

### Participants

The PISA 2018 survey was conducted by the Organisation for Economic Cooperation and Development (OECD). We used the PISA survey data collected between March 2018 and August 2018. More than 600,000 adolescents aged 15–16 years attending secondary education participated worldwide in the 2018 survey [21]. In recent years, the OECD has put more focus on carrying out research on adolescents’ wellbeing (e.g., life satisfaction) and social factors (e.g., exposure to bullying). PISA 2018 adopted a two-stage stratified sample design where schools were sampled systematically with probabilities proportional to the number of adolescents enrolled in the school. The second stage involved adolescents being randomly sampled within those schools and weights allocated to ensure the surveyed sample was representative of adolescents in the population. More details on PISA 2018 and the sampling method can be found in the technical report and user guide [21]. In the present sample, we excluded 10 countries due to missingness of the outcome and/or the exposure variables of interest. We excluded 7 cities and economic regions to ensure cross-national comparisons. In total, we excluded 17 countries resulting in a sample comprising of 479,685 adolescents from 63 countries. A participant flow diagram is given in Supplemental Fig. 1.

### Data and measures

#### Outcome variables – psychological distress and life satisfaction

Psychological distress was measured using 4 items that asked the adolescents how often they felt (1) sad, (2) miserable, (3) scared, or (4) afraid on a 4-point frequency scale that ranged from never to always. We included adolescents who answered a total of 2 or more items. Mean imputation was applied for missing items. The overall score for psychological distress ranged from 4 to 16 after multiplying the score by 4 to give the original range. Life satisfaction was assessed with the question *‘overall, how satisfied are you with your life as a whole these days?’*. Life satisfaction was measured on a frequency scale from 0 to 10 where 0 was ‘not at all satisfied’ and 10 ‘completely satisfied’. We excluded adolescents who did not answer the life satisfaction question.

#### Exposure variable – bullying victimisation

Adolescents were asked *‘During the past 12 months, how often have you had the following experiences in school? (some experiences can also happen in social media)’*. Adolescents completed six items including *‘I got hit or pushed around by other students’*, *‘I was threatened by other students’* and *‘Other students left me out of things on purpose’*. The six items were grouped into corresponding subtypes to allow analysis by subtypes (full set of bullying items and subtypes are given in Supplemental Table 1).

Frequency was assessed on a 4-point scale: never or almost never (1), a few times a year (2), a few times a month (3), and once a week or more (4). We included adolescents who answered 3 or more items out of 6 for the total scale, and at least 1 out of 2 items per subtype i.e., those that answered less than 50% were excluded. Mean imputation was used for missing items. To create the ‘total bullying victimisation’ (hereafter referred to as ‘bullying victimisation’) score, responses to all six options were summed (range: 6–24). To create scores for each subtype, the two items were summed (range: 2–8). All sum scores were standardised to a mean of zero and a standard deviation of one.

### Country-level factors:

#### Bullying prevalence

We defined high bullying prevalence as exposure to bullying a few times a month and/or once a week or more [22]. A binary variable was created for each respondent (i.e., bullied or not bullied). The mean prevalence score was calculated per country.

#### Income inequality

We used the Gini index as the country-level income inequality predictor for this study (henceforth, we use income inequality and the Gini index interchangeably). The Gini index is a widely used measure of inequality in income distribution and ranges from 0 (complete equality) to 100 (complete inequality). For countries with missing Gini index values in the year 2018, we used available data within a 10-year period (2008–2018), retrieved from the World Bank dataset on the 13th January 2023 [23].

#### National wealth

We used the Gross domestic product per capita based on purchasing power parity (henceforth GDP) as the national wealth indicator for this study. GDP was selected as the best means of comparing country wealth as it accounts for differences in price levels between countries [24]. For each country included in this study, we sourced the GDP from the World Bank 2018 dataset, retrieved 13th January 2023 [23].

The sample characteristics are given in Supplemental Table 2.

#### Covariates

Gender (girl = 0, boy = 1) was adjusted for as there are gender differences in both bullying victimisation [25] and mental health [26].

Socioeconomic status was adjusted for using the Economic Social and Cultural Status (ESCS) PISA index variable derived from measures of parental education, highest parental occupation and home possessions [21]. There is evidence to suggest associations between increased likelihood of bullying for both low parental education [27] and low parental occupation [28] during childhood and adolescence. Research also reports associations between mental health problems in children and adolescents and low socioeconomic status, indicated by household income, parental education and parental unemployment [29].

### Analyses

We conducted multilevel regression analyses within the statistical software R version 4.1.2, package *lme4* [30]. The PISA-recommended weight variable ‘SENWT’ was used to allow inferences to the target population in each country.

Supplemental Table 3 details the multi-level modelling approach taken to investigate how the relationships between bullying victimisation, psychological distress and life satisfaction vary between countries. Gender and ESCS were controlled for in all models. Firstly, a Baseline Model was created which included only the outcome variable and covariates. This model acted as a reference to estimate the magnitude of variation at each level. We used the Baseline Models for each outcome to calculate the Intraclass Correlation Coefficient (i.e. the proportion of the total variance explained by the variation between countries) [31]. For psychological distress, 7.8% of the variation was explained by cross-country variation and for life satisfaction this variation was 5.9%. In Model 2, bullying victimisation was added as a predictor. This model tested the fixed effects of bullying victimisation on psychological distress and life satisfaction. Bullying victimisation was added as a random slope in Model 3. We tested the significance of the random slope by comparing the model fit between Model 2 and Model 3 (i.e., with or without a random slope) using a log-likelihood difference test. This tested the hypothesis that the strength of the associations between bullying victimisation and poor mental health varies across countries. We included the moderators (i.e., country-level factors) in Models 4, 5 and 6. In Model 4, we included bullying prevalence as a fixed main effect and an interaction term with bullying victimisation. Models 5 (the Gini index) and 6 (GDP) took the same approach.

## Results

### Fixed and random effects of bullying victimisation

Higher levels of bullying victimisation were associated with higher psychological distress (β = 0.181; 95%CI: 0.178, 0.184) and lower life satisfaction (β = -0.158; 95%CI: -0.162, -0.155) in adolescents (Supplemental Tables 4–6). For both psychological distress and life satisfaction, adding a random effect for bullying victimisation, i.e., allowing the slope to vary by country, improved the fit of the model.

**Fig. 1:**
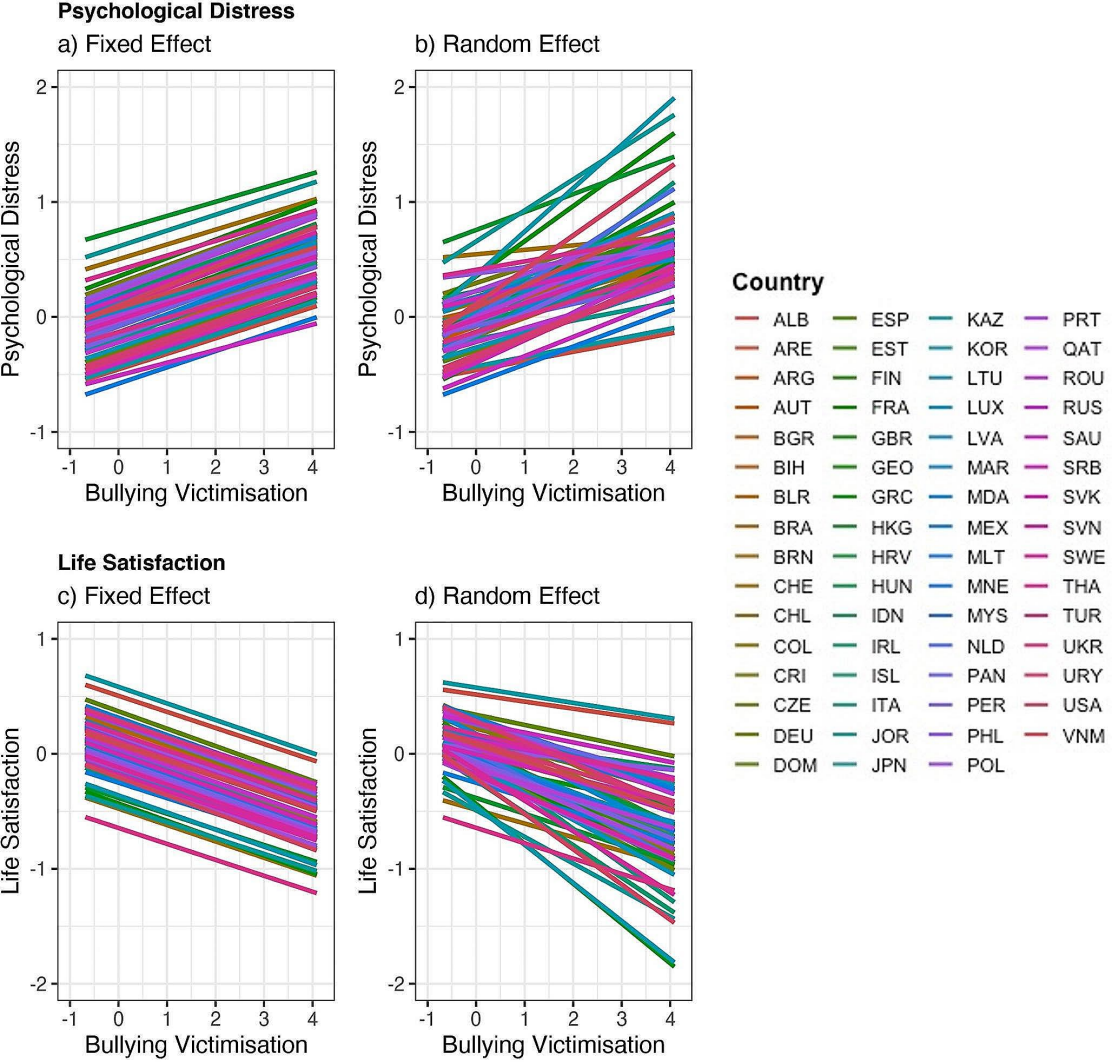
Fixed and Random Effects of Bullying Victimisation on Psychological Distress and Life Satisfaction. **a**) Fixed effect of bullying victimisation on psychological distress. Parallel slopes indicate a fixed effect i.e., the effect size was constant across countries. **b**) Random effect of bullying victimisation on psychological distress. Bullying victimisation had varying effects on psychological distress as seen by the varying slopes in the right panel. Countries varied in the way bullying victimisation affected psychological distress. Similar pattern of results are shown in **c**) and **d**) for life satisfaction. Country full names are given in Supplemental Table 2

**Fig. 2:**
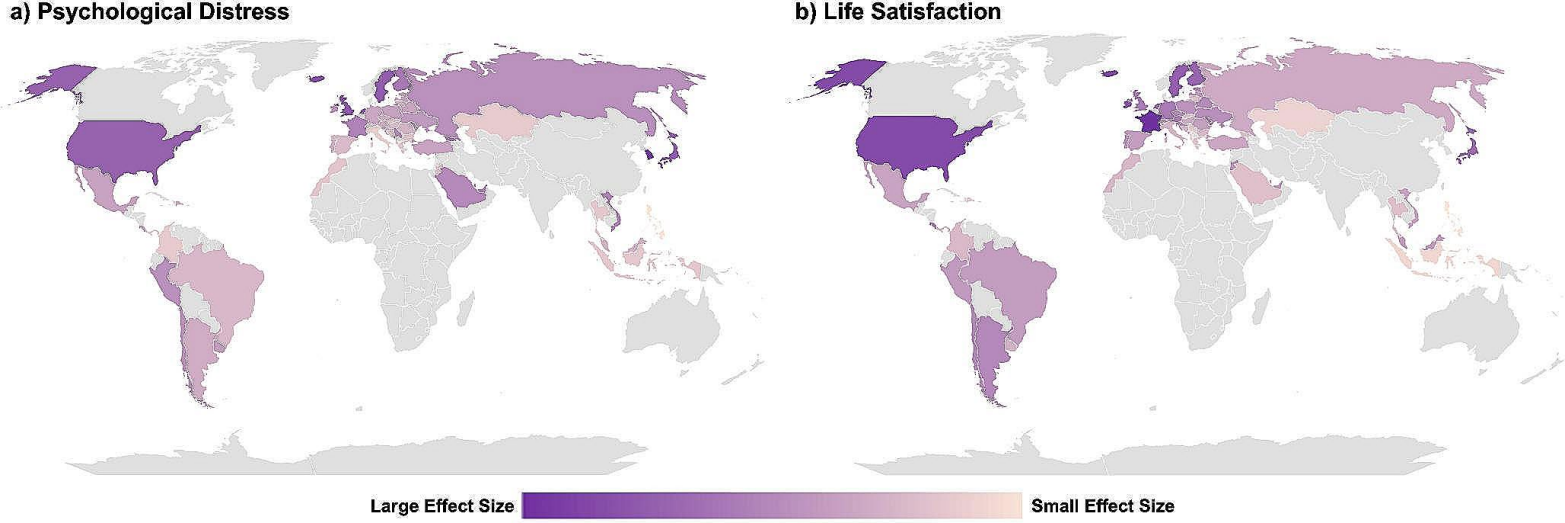
The Random Effects of Bullying Victimisation on Psychological Distress and Life Satisfaction. We modelled the regression coefficients for the random effects of bullying victimisation into a map chart for **(a)** psychological distress and **(b)** life satisfaction. Larger effect sizes, i.e., darker purple shading, meant that bullying victimisation had a stronger effect on the mental health outcome in that country. Note, countries in grey were not included in the analytical sample. The countries included in the sample and the regression coefficients can be found in Supplemental Table 2

Plotted in Fig. 1 are the fixed and random effects of bullying victimisation on mental health outcomes. In the fixed effect models (Fig. 1a and c), the effect sizes of the associations between bullying victimisation and mental health outcomes were the same across countries (resulting in parallel slopes). The random effect models (Fig. 1b and d) allowed each country to have its own regression slope. The country-specific effects are plotted in Fig. 2, shown as a world map. The effects ranged from β = 0.08 in the Philippines to β = 0.40 in South Korea for psychological distress and from β = −0.05 in the Philippines to β = −0.36 in the United Kingdom for life satisfaction.

Adding a random effect for physical, verbal and relational bullying victimisation improved the fit of the model. The random effects of physical, verbal and relational bullying on psychological distress and life satisfaction yielded associations of similar magnitude to overall bullying victimisation. Relational bullying victimisation had the highest negative association on both psychological distress (β = 0.188; 95%CI: 0.187, 0.188) and life satisfaction (β = -0.175; 95%CI: -0.175, -0.174). Whereas physical bullying victimisation had the lowest association on both psychological distress (β = 0.130; 95%CI: 0.130, 0.131) and life satisfaction (β = -0.111; 95%CI: -0.111, -0.111) (Supplemental Tables 7–8). Across countries, psychological distress was negatively associated most in Iceland (β = 0.257) for physical bullying, in the United States (β = 0.285) for verbal bullying, and in Korea (β = 0.341) for relational bullying. Life satisfaction was negatively associated most in the United Kingdom (β = -0.267, β = -0.331) for both physical and relational bullying respectively, and the United States (β = -0.274) for verbal bullying (Supplemental Table 9).

### Moderating effects of country-level factors

Within the random effects model, we tested the interactions between bullying victimisation and three moderators – bullying prevalence, Gini index and GDP (Tables 1 and 2). Bullying prevalence significantly moderated the associations between bullying victimisation and both psychological distress and life satisfaction (*p* < 0.001 for both mental health measures). We detected no significant moderating effect of the Gini index but GDP had significant main effects and moderating effects on both psychological distress and life satisfaction.

**Table 1:** Moderating effects of bullying prevalence, the Gini index and GDP (country-level factors) on psychological distress. All three country-level factors were included as a main effect and as an interaction term with bullying victimisation

	Models^a^		
	Bullying Prevalence	Gini index	GDP
Bullying Victimisation	0.304^b^	0.268	0.158
	(0.258, 0.350)^c^	(0.181, 0.355)	(0.127, 0.188)
	***p*** **= 1e-22**^d^	***p*** **= 1.56e-09**	***p*** **= 1e-22**
Bullying Prevalence	0.141		
	(-0.583, 0.866)		
	*p* = 7.02e-01		
Bullying Victimisation* Bullying Prevalence	-0.391		
	(-0.548, -0.235)		
	***p*** **= 8.96e-07**		
Gini index^e^		0.0004	
		(-0.008, 0.009)	
		*p* = 9.24e-01	
Bullying Victimisation*Gini index		-0.002	
		(-0.005, 0.0003)	
		*p* = 8.27e-02	
GDP^f^			0.032
			(0.001, 0.062)
			***p*** **= 4.17e-02**
Bullying Victimisation *GDP			0.011
			(0.003, 0.018)
			***p*** **= 5.31e-03**

^a^All three multilevel linear regression models were adjusted for gender and parental economic, social cultural status (ESCS). All models were weighted. Asterisk (*) indicate an interaction term

^b^Regression coefficients with estimates

^c^95% confidence intervals upper and lower limits

^d^*p* values displayed. Bold *p* indicated significant value

^e^The Gini index and GDP for each country included in this analysis were sourced from the World Bank 2018 dataset

^f^GDP indicated GDP per capita purchasing power parity

**Table 2:** Moderating effects of bullying prevalence, the Gini index and GDP (country-level factors) on life satisfaction. All three country-level factors were included as a main effect and as an interaction term with bullying victimisation

	Models^a^		
	Bullying Prevalence	Gini index	GDP
Bullying Victimisation	-0.289^b^	-0.265	-0.122
	(-0.335, -0.244)^c^	(-0.355, -0.174)	(-0.151, -0.094)
	***p*** **= 1e-22**^d^	***p*** **= 9.32e-09**	***p*** **= 1e-22**
Bullying Prevalence	0.340		
	(-0.300, 0.979)		
	*p* = 2.98e-01		
Bullying Victimisation* Bullying Prevalence	0.413		
	(0.260, 0.567)		
	***p*** **= 1.36e-07**		
Gini index^e^		0.003	
		(-0.005, 0.012)	
		*p* = 4.08e-01	
Bullying Victimisation*Gini index		0.003	
		(-0.00001, 0.005)	
		*p* = 5.07e-02	
GDP^f^			-0.053
			(-0.077, -0.028)
			***p*** **= 2.43e-05**
Bullying Victimisation *GDP			-0.015
			(-0.022, -0.008)
			***p*** **= 3.10e-05**

^a^All three multilevel linear regression models were adjusted for gender and parental economic, social cultural status (ESCS). All models were weighted. Asterisk (*) indicate an interaction term

^b^Regression coefficients with estimates

^c^95% confidence intervals upper and lower limits

^d^*p* values displayed. Bold *p* indicated significant value

^e^The Gini index and GDP for each country included in this analysis were sourced from the World Bank 2018 dataset

^f^GDP indicated GDP per capita purchasing power parity

**Fig. 3:**
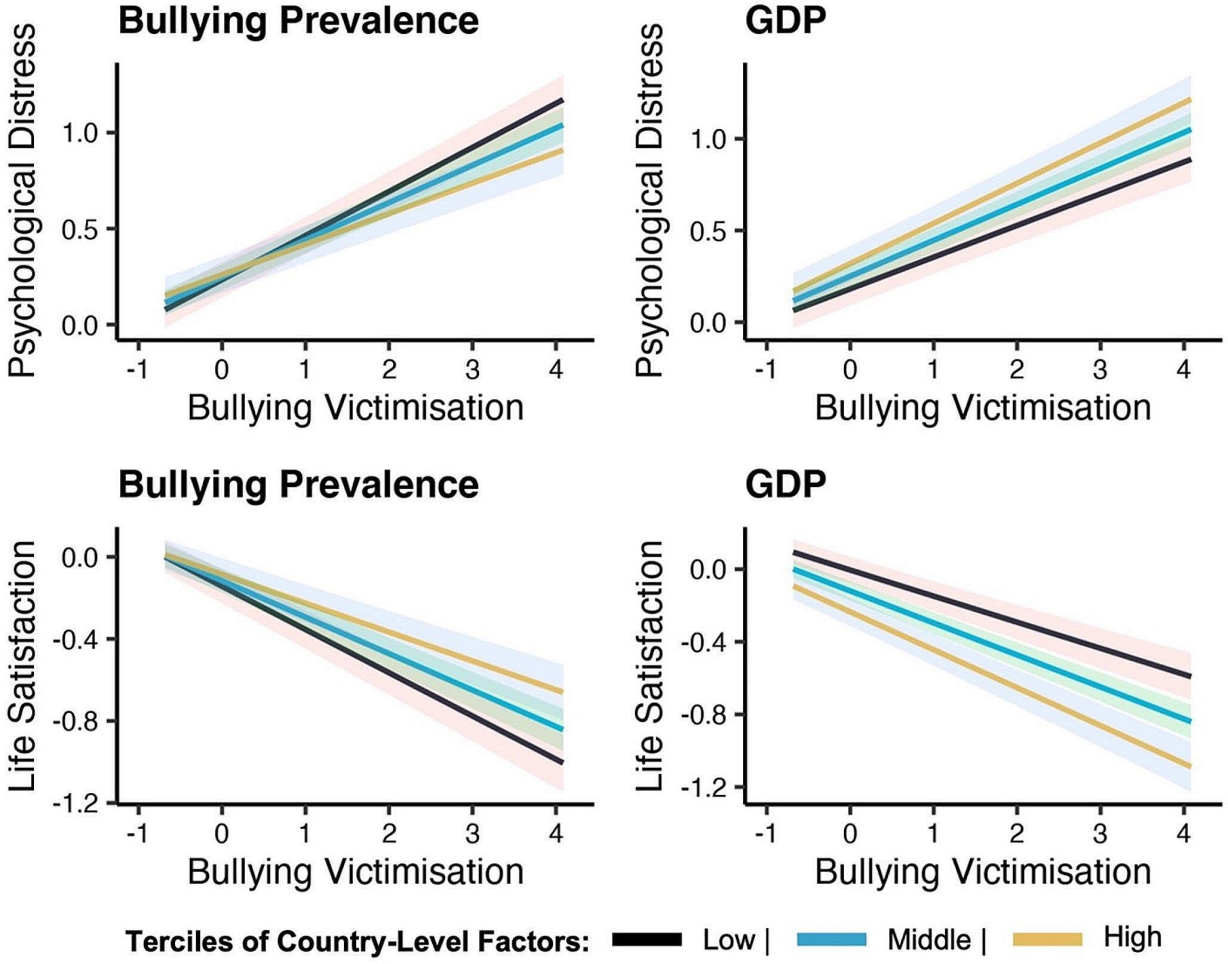
Interaction Regression Plots. Here we illustrated the significant interactions from Tables 1 and 2. We used the ‘*quantile*’ function in R to create the low, middle and high terciles for the moderators. For illustration, we took the median scores of each tercile at 16.7%, 50% and 83.3% to represent the low, middle and high terciles, respectively. For mean bullying prevalence this was 0.19, 0.26, 0.34; and for GDP this was 1.44, 3.11 and 5.52. These scores were used to illustrate the terciles thresholds for each moderator

To illustrate the moderating effects of bullying prevalence and GDP, we plotted the associations between bullying victimisation and mental health outcomes for the low, middle and high terciles of each moderator (Fig. 3). The association between bullying victimisation, higher psychological distress and lower levels of life satisfaction was larger in countries where bullying prevalence was low. In high-income countries, there were stronger associations between bullying victimisation, higher psychological distress and lower life satisfaction, when compared to the associations in low-income countries.

For each subtype of bullying victimisation, the moderating effects of bullying prevalence and GDP on psychological distress and life satisfaction yielded similar results to those for the overall score of bullying victimisation (Supplemental Tables 10–15). In contrast, the Gini index significantly moderated the associations between relational bullying victimisation and both outcomes (Supplemental Tables 12 and 15).

## Discussion

We first examined whether the associations between bullying victimisation, psychological distress and life satisfaction in adolescents varied across countries. We observed that bullying victimisation was detrimental to mental health in all countries and the strength of the associations varied substantially across countries. Secondly, we tested the moderating role of country-level factors in these associations. Bullying prevalence and GDP had significant moderating effects for both psychological distress and life satisfaction. Associations were larger between bullying victimisation and poorer mental health outcomes in countries with low bullying prevalence and in high income countries. We also found that relational bullying victimisation had the greatest negative effects on both psychological distress and life satisfaction across countries. The Gini index had significant moderating effects for associations between relational bullying victimisation and both mental health outcomes.

Our results confirmed associations between bullying victimisation and poor mental health outcomes in adolescents across all included countries which mirrors findings in the literature [32]. Although direction of associations (i.e., greater bullying associated with higher psychological distress and lower life satisfaction) remained consistent in all 63 countries, the magnitude of the association that bullying victimisation had on mental health varied. We also confirmed that verbal bullying victimisation had a greater negative effect on adolescent mental health than physical bullying victimisation across countries [33], however we found that relational bullying had the greatest negative effects on both mental health outcomes.

We examined whether the country-level factors we tested had moderating effects on the associations between bullying victimisation and mental health outcomes. For countries that had lower bullying prevalence, the association between being bullied and higher psychological distress or lower life satisfaction was stronger than in countries where bullying prevalence was higher.

This finding is consistent with research suggesting that being bullied has greater negative effects on adolescents’ mental wellbeing in schools where bullying is infrequent [14–16]. A previous cross-country study suggested that the relationship between bullying victimisation and wellbeing was stronger in schools and countries where bullying is less frequent [34].

Here, we formally tested this moderating effect across a wider range of countries, demonstrating that when the country-level prevalence of bullying is low, the association with mental health is stronger. Taken together, these findings provide evidence that the healthy context paradox, which has been observed for bullying at classroom or school-level [16, 35] also applies at country-level. When the prevalence of bullying decreases, either naturally or following interventions, mental health in the remaining victims appears degraded. Such findings can be interpreted in, at least, two non-exclusive ways. First, in contexts where being bullied is or become less normative, children experiencing bullying may be singled out more, leading to more damaging consequences for mental health. Second, those who still experience bullying victimisation in such contexts may have pre-exiting vulnerabilities, including mental health difficulties, that may contribute to explain why they experience more or heightened mental health adverse outcomes [36, 37].

To test the healthy context paradox further, we examined other potential country-level moderators, including GDP. Our results showed that in high-income countries, there was higher psychological distress and lower life satisfaction among bullied victims. A previous cross-country study has shown that the prevalence of bullying is lower in high-income countries [38]. Consistent with the healthy context paradox framework, such reduced levels of bullying victimisation may lead to increased psychological impacts of bullying.

We found that the Gini index did not moderate the associations between overall bullying victimisation and mental health outcomes. It is possible that school-level inequality or inequality as perceived by individual children may matter more [39] than country-level inequality. That said, we still observed a significant interaction between the Gini index and relational bullying victimisation. Considering that the magnitude of the associations between relational bullying victimisation and mental health outcomes was larger than for other subtypes, the moderating effect of the Gini index may have been more easily detectable for relational bullying victimisation compared to other subtypes.

The variation in the association between bullying victimisation and adolescents’ mental health may stem from differences in sociocultural environments such as family, schools and social groups [40, 41]. Such environments may uphold different behavioural and social norms, potentially shaping adolescents’ attitudes and expectations towards bullying and, in turn, how it influences mental health [41–43]. Further research, within and across countries, should focus on the mechanisms that contribute to explain the healthy country paradox, which would provide additional insights on how best to shape interventions aiming to reduce bullying victimisation and support victims.

### Limitations

Limitations of this study should be noted. First, although the bullying victimisation item specified that ‘*some experiences can also happen in social media*,’ there was no stand-alone questionnaire item for cyberbullying/cyber-victimisation in the survey. Second, we were limited in the number of country-level contextual markers that were measured. Third, there are no participating countries in the PISA study from the African region as classified by the World Bank [23].

## Conclusions

This multi-country investigation found that among adolescents, bullying victimisation was significantly associated with higher psychological distress and lower life satisfaction, with varying effects across countries. Country-level factors such as bullying prevalence and GDP, significantly moderated the associations between bullying victimisation, higher psychological distress and lower life satisfaction. In line with the healthy context paradox framework, the negative association bullying victimisation had on mental health outcomes was larger in countries where the prevalence of bullying was lower, as well as in higher-income countries.

Current anti-bullying programs considers whole-school interventions such as curriculum-based approaches or management strategies (i.e., teacher training) and the school environment, and less focus is on the individuals that are bullied. When designing bullying interventions, the focus cannot only be on reducing bullying prevalence, but additional measures should be taken for the children left behind, who are still bullied and may be at heightened risk of adverse mental health outcomes.

## Electronic supplementary material

Below is the link to the electronic supplementary material.

Supplementary Material 1

## Data Availability

The data that supports the findings of this study is available online and can be downloaded from the Programme for International Student Assessment (PISA) 2018 survey database at https://www.oecd.org/pisa/data/2018database/.
