# Supplementary material for: The healthy context paradox: a cross-country analysis of the association between bullying victimisation and adolescent mental health
Source: Eur Child Adolesc Psychiatry. 2024 Jun 4;34(1):215–24. doi: 10.1007/s00787-024-02483-x (PMC11805777; doi:10.1007/s00787-024-02483-x)
Supplement: Supplementary file 1 — Supplementary Material 1 [file 787_2024_2483_MOESM1_ESM.pdf]

SUPPLEMENTARY INFORMATION

Supplementary Figure 1. Participant Flow Diagram ____ 2

Supplementary Table 1. Bullying Victimisation Items. Survey questionnaire includes 6 items grouped into physical, verbal and relational bullying subtypes. ____ 3

Supplementary Table 2. Sample Summary Characteristics ____ 4

Supplementary Table 3. Models fitted for psychological distress and life satisfaction in multi-level modelling analyses. ____ 7

Supplementary Table 4. Effect of bullying victimisation on psychological distress and life satisfaction in the fixed model (Model 2) ____ 8

Supplementary Table 5. Effect of bullying victimisation on psychological distress and life satisfaction in the random model (Model 3) ____ 8

Supplementary Table 6. Comparison between the fixed and random effects models of bullying victimisation on psychological distress and life satisfaction ____ 9

Supplementary Table 7. Comparison between the fixed and random effects models of physical, verbal and relational bullying victimisation on psychological distress and life satisfaction ____ 10

Supplementary Table 8. Random effects of physical, verbal and relational victimisation on psychological distress and life satisfaction ____ 11

Supplementary Table 9. Regression coefficients from the random effects of physical, verbal and relational victimisation on psychological distress and life satisfaction ____ 12

Supplementary Table 10. Moderating effects of country-level factors on the association between physical bullying victimisation and psychological distress ____ 15

Supplementary Table 11. Moderating effects of country-level factors on the association between verbal bullying victimisation and psychological distress ____ 16

Supplementary Table 12. Moderating effects of country-level factors on the association between relational bullying victimisation and psychological distress ____ 17

Supplementary Table 13. Moderating effects of country-level factors on the association between physical bullying victimisation and life satisfaction ____ 18

Supplementary Table 14. Moderating effects of country-level factors on the association between verbal bullying victimisation and life satisfaction ____ 19

Supplementary Table 15. Moderating effects of country-level factors on the association between relational bullying victimisation and life satisfaction ____ 20

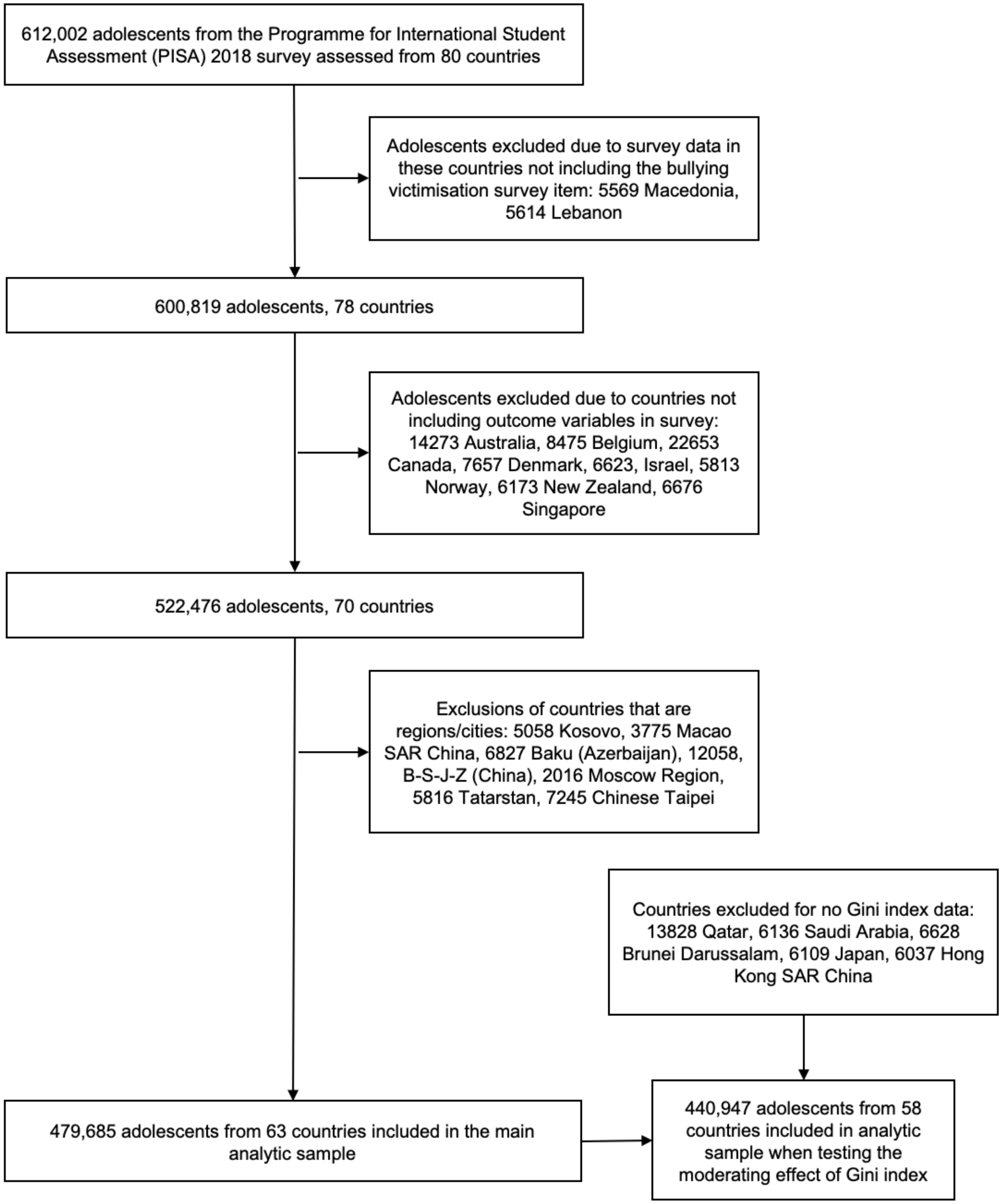

Supplementary Figure 1. Participant Flow Diagram. Adolescents were aged 15-16 years and survey data was collected between March 2018-August 2018. Outcome variables were psychological distress and life satisfaction. B-S-J-Z is an acronym for the four Chinese provinces that participated: Beijing, Shanghai, Jiangsu and Zhejiang.

Supplementary Table 1. Bullying Victimisation Items. Survey questionnaire includes 6 items grouped into physical, verbal and relational bullying subtypes.

During the past 12 months, how often have you had the following experiences in school? (*some experiences can also happen in social media*)

Bullying Victimisation Items	***Subtype***
1) I got hit or pushed around by other students<br>2) Other students took away or destroyed things that belonged to me	Physical
3) I was threatened by other students<br>4) Other students made fun of me	Verbal
5) Other students left me out of things on purpose<br>6) Other students spread nasty rumors about me	Relational

Supplementary Table 2. Sample Summary Characteristics.

Region	Country Code	Country Name	Number of Participants	Percentage of Girls per Country (%)	Psychological Distress Coefficient[a]	Life Satisfaction Coefficient[b]	Mean Bullying Prevalence[c] (%)	Gini index[d]	GDP per capita[e] (millions, $)
**Eastern Mediterranean Region**	ARE	United Arab Emirates	19277	48.7	0.242	-0.169	33.2	26.0	71551
	JOR	Jordan	8963	51.5	0.142	-0.178	37.9	33.7	9585
	MAR	Morocco	6814	47.9	0.130	-0.125	47.4	39.5	8072
	QAT	Qatar	13828	50.3	0.237	-0.147	34.0		91417
	SAU	Saudi Arabia	6136	48.8	0.239	-0.113	30.4		45841
**Region of the Americas**	ARG	Argentina	11975	52.0	0.176	-0.205	31.7	41.3	22747
	BRA	Brazil	10691	51.2	0.156	-0.171	29.7	53.9	14620
	CHL	Chile	7621	50.0	0.220	-0.199	23.7	44.4	25072
	COL	Colombia	7522	51.3	0.124	-0.122	31.9	50.4	14426
	CRI	Costa Rica	7221	50.1	0.190	-0.217	24.6	48.0	20503
	DOM	Dominican Republic	5674	50.9	0.176	-0.095	48.6	43.7	17484
	MEX	Mexico	7299	52.4	0.194	-0.158	23.1	46.7	20278
	PAN	Panama	6270	50.6	0.121	-0.124	36.2	49.2	31126
	PER	Peru	6086	49.3	0.227	-0.176	23.4	42.4	12696
	URY	Uruguay	5263	51.9	0.208	-0.137	27.7	39.7	23185
	USA	United States	4838	49.1	0.311	-0.309	26.4	41.4	61356
**South-East Asian Region**	IDN	Indonesia	12098	51.6	0.126	-0.072	40.5	38.4	1397
	THA	Thailand	8633	54.4	0.126	-0.113	26.9	36.4	17669
**Western Pacific Region**	BRN	Brunei Darussalam	6828	49.5	0.091	-0.135	50.8		59650
	JPN	Japan	6109	51.1	0.318	-0.252	17.3		41739

Region	Country Code	Country Name	Number of Participants	Percentage of Girls per Country (%)	Psychological Distress Coefficient	Life Satisfaction Coefficient	Mean Bullying Prevalence (%)	Gini index	GDP per capita (millions, $)
	MYS	Malaysia	6111	51.2	0.164	-0.170	36.0	41.1	26836
	PHL	Philippines	7233	53.5	0.076	-0.051	65.8	42.3	8366
	VNM	Vietnam	5377	51.7	0.245	-0.159	27.0	35.7	9636
	HKG	Hong Kong SAR, China	6037	48.9	0.214	-0.159	29.7		61055
	KOR	South Korea	6650	48.0	0.403	-0.341	9.4	31.4	41966
**European Region**	ALB	Albania	6359	49.8	0.126	-0.080	26.3	30.1	13319
	AUT	Austria	6802	48.8	0.169	-0.220	23.4	30.8	55217
	BGR	Bulgaria	5294	47.8	0.134	-0.123	35.3	41.3	22210
	BIH	Bosnia and Herzegovina	6480	48.6	0.132	-0.125	26.2	33.0	14067
	BLR	Belarus	5803	47.8	0.189	-0.201	19.1	25.2	18975
	CHE	Switzerland	5822	47.9	0.143	-0.188	23.3	33.1	70559
	CZE	Czech Republic	7019	50.1	0.166	-0.196	28.1	25.0	39941
	DEU	Germany	5451	46.3	0.187	-0.226	23.1	31.7	53431
	ESP	Spain	35943	50.0	0.152	-0.168	16.4	34.7	40255
	EST	Estonia	5316	49.9	0.189	-0.198	25.7	30.3	34980
	FIN	Finland	5649	49.1	0.270	-0.265	18.0	27.3	48049
	FRA	France	6308	48.8	0.251	-0.222	21.8	32.4	45246
	GBR	United Kingdom	13818	50.6	0.326	-0.355	27.2	35.1	46878
	GEO	Georgia	5572	48.1	0.257	-0.111	24.8	36.4	14253
	GRC	Greece	6403	49.6	0.135	-0.104	27.2	32.9	29141
	HRV	Croatia	6609	50.1	0.162	-0.189	18.9	29.7	28225
	HUN	Hungary	5132	50.8	0.146	-0.143	22.4	29.6	31123
	IRL	Ireland	5577	49.8	0.215	-0.293	23.3	30.6	83340
	ISL	Iceland	3296	50.2	0.342	-0.312	18.9	26.1	56816
	ITA	Italy	11785	48.2	0.114	-0.128	23.3	35.2	42046
	KAZ	Kazakhstan	19507	49.1	0.122	-0.080	31.0	27.8	25544

Region	Country Code	Country Name	Number of Participants	Percentage of Girls per Country (%)	Psychological Distress Coefficient	Life Satisfaction Coefficient	Mean Bullying Prevalence (%)	Gini index	GDP per capita (millions, $)
	LTU	Lithuania	6885	49.0	0.171	-0.111	23.8	35.7	35447
	LUX	Luxembourg	5230	49.6	0.210	-0.236	21.1	35.4	114165
	LVA	Latvia	5303	50.6	0.184	-0.173	36.3	35.1	30051
	MDA	Republic of Moldova	5367	48.8	0.228	-0.209	24.3	25.7	12368
	MLT	Malta	3363	47.9	0.169	-0.152	32.3	28.7	44616
	MNE	Montenegro	6666	48.6	0.201	-0.131	25.8	36.8	20690
	NLD	Netherlands	4765	48.9	0.299	-0.240	12.6	28.7	56061
	POL	Poland	5625	50.8	0.195	-0.188	26.6	30.2	31739
	PRT	Portugal	5932	49.6	0.172	-0.183	14.1	33.5	34041
	ROU	Romania	5075	48.2	0.164	-0.165	34.2	35.8	28741
	RUS	Russian Federation	7608	50.7	0.229	-0.148	38.2	37.5	26656
	SRB	Serbia	6609	49.5	0.231	-0.092	26.0	35.0	17456
	SVK	Slovak Republic	5965	50.3	0.129	-0.112	28.8	25.0	31230
	SVN	Slovenia	6401	46.8	0.187	-0.196	22.8	24.6	37996
	SWE	Sweden	5504	50.2	0.319	-0.275	19.7	30.0	52349
	TUR	Turkey	6890	49.3	0.193	-0.153	24.7	41.9	28158
	UKR	Ukraine	5998	47.6	0.210	-0.193	22.6	26.1	12337

[a b] The psychological distress and life satisfaction coefficients were the standardised regression coefficients corresponding to the regression of psychological distress and life satisfaction on bullying victimisation, as extracted from Model 3.

[c] Bullying prevalence indicated exposure to bullying a few times a month and/or once a week or more. The mean was calculated per country.

[d] Countries with no Gini index value had no documented data between 2008-2018 on World Bank Data, retrieved 13th January 2023.

[e] GDP (purchasing power parity) values were taken from the World Bank 2018 data, retrieved 13th January 2022.

Supplementary Table 3. Models fitted for psychological distress and life satisfaction in multi-level modelling analyses.

Model		Fixed Effects	Random Effects
1)	Baseline	Gender<br>ESCS	Country
2)	Effects of Bullying Victimisation: Fixed Model	Gender<br>ESCS<br>Bullying Victimisation	Country
3)	Effects of Bullying Victimisation: Random Model	Gender<br>ESCS<br>Bullying Victimisation	Bullying|Country
4)	Moderating effect of bulling prevalence	Gender<br>ESCS<br>Bullying Victimisation*Bullying prevalence	Bullying|Country
5)	Moderating effect of Gini index	Gender<br>ESCS<br>Bullying Victimisation*Gini index	Bullying|Country
6)	Moderating effect of GDP	Gender<br>ESCS<br>Bullying Victimisation*GDP	Bullying|Country

Note: All models controlled for gender and ESCS. The Baseline model was used to calculate the intraclass correlation coefficient. In models 4-6, Bullying prevalence, Gini index and GDP were the country-level factors. Models 3-6 were used to test the random effects of the subtypes of bullying where ‘Bullying Victimisation’ was changed to physical, verbal and relational as per analysis. ‘ESCS’ referred to *Economic, Social and Cultural Status*. ‘Bullying|Country’ referred to the random slope for the varied effect of bullying victimisation in each country where ‘Bullying’ referred to Bullying Victimisation.

Supplementary Table 4. Effect of bullying victimisation on psychological distress and life satisfaction in the fixed model (Model 2).

**Psychological Distress**			
	**Estimate**	**95% CI**	***p value***
Gender	0.502	(0.496, 0.508)	**7.99e-14**
ESCS	0.031	(0.027, 0.034)	**1e-22**
Bullying Victimisation	0.195	(0.178, 0.212)	**1e-22**
**Life Satisfaction**			
Gender	-0.207	(-0.213, -0.201)	**1.72e-03**
ESCS	0.042	(0.038, 0.045)	**0.000**
Bullying Victimisation	-0.174	(-0.191, -0.157)	**0.000**

Note: CI = Confidence Interval. ESCS = Economic, Social and Cultural Status.

Supplementary Table 5. Effect of bullying victimisation on psychological distress and life satisfaction in the random model (Model 3).

**Psychological Distress**			
	**Estimate**	**95% CI**	***p value***
Gender	0.502	(0.496, 0.508)	**1e-22**
ESCS	0.031	(0.028, 0.035)	**1e-22**
Bullying Victimisation	0.181	(0.178, 0.184)	**1e-22**
**Life Satisfaction**			
Gender	-0.209	(-0.215, -0.203)	**0.000**
ESCS	0.042	(0.038, 0.045)	**0.000**
Bullying Victimisation	-0.158	(-0.162, -0.155)	**0.000**

Note: CI = Confidence Interval. ESCS = *Economic, Social and Cultural Status.*

Supplementary Table 6. Comparison between the fixed and random effects models of bullying victimisation on psychological distress and life satisfaction.

	npar	AIC	BIC	logLik	deviance	Chisq	Df	*p value*
**Psychological Distress**								
**Fixed**	6	1083918	1083983	-541953.0	1083906			
**Random**	8	1082574	1082660	-541278.9	1082558	1348.355	2	**0.000**
**Life Satisfaction**								
**Fixed**	6	1095703	1095768	-547845.3	1095691			
**Random**	8	1094334	1094421	-547159.1	1094318	1372.434	2	**0.000**

Note: A one-way ANOVA was used to statistically compare the means between both models. npar = Number of parameters; AIC = Akaike's Information Criteria; BIC = Bayesian Information Criteria; logLik = Log-Likelihood; Chisq = Chisquared; Df = Degrees of Freedom. Bold *p* value indicated statistically significant *p* value.

Supplementary Table 7. Comparison between the fixed and random effects models of physical, verbal and relational bullying victimisation on psychological distress and life satisfaction.

	npar	AIC	BIC	logLik	deviance	Chisq	Df	*p value*
**Psychological Distress**								
Physical Bullying Victimisation								
**Fixed**	6	1091207	1091272	-545598	1091195			
**Random**	8	1090500	1090587	-545242	1090484	711.5	2	**0.000**
Verbal Bullying Victimisation								
**Fixed**	6	1086064	1086129	-543026	1086052			
**Random**	8	1084961	1085047	-542472	1084945	1107.3	2	**0.000**
Relational Bullying Victimisation								
**Fixed**	6	1086698	1086761	-543342	1086684			
**Random**	8	1085667	1085754	-542826	1085651	1032.9	2	**0.000**
**Life Satisfaction**								
Physical Bullying Victimisation								
**Fixed**	6	1101536	1101601	-550762	1101524			
**Random**	8	1100954	1101041	-550469	1100938	585.95	2	**0.000**
Verbal Bullying Victimisation								
**Fixed**	6	1099033	1099098	-549511	1099021			
**Random**	8	1097988	1098075	-548986	1097971	1049.3	2	**0.000**
Relational Bullying Victimisation								
**Fixed**	6	1099117	1099182	-549552	1099105			
**Random**	8	1097901	1097988	-548943	1097885	1219.3	2	**0.000**

Note: A one-way ANOVA was used to statistically compare the means between both models. npar = Number of parameters; AIC = Akaike's Information Criteria; BIC = Bayesian Information Criteria; logLik = Log-Likelihood; Chisq = Chisquared; Df = Degrees of Freedom. Bold *p* value indicated statistically significant *p* value.

Supplementary Table 8. Random effects of physical, verbal and relational victimisation on psychological distress and life satisfaction.

**Psychological Distress**			
	**Estimate**	**95% CI**	***p value***
Physical Bullying	0.130	(0.130, 0.131)	**0.000**
Verbal Bullying	0.186	(0.186, 0.187)	**0.000**
Relational Bullying	0.188	(0.187, 0.188)	**0.000**
**Life Satisfaction**			
Physical Bullying	-0.111	(-0.111, -0.111)	**0.000**
Verbal Bullying	-0.160	(-0.160, -0.159)	**0.000**
Relational Bullying	-0.175	(-0.175, -0.174)	**0.000**

Note: Models were adjusted for gender and parental economic, social cultural status (ESCS). CI = Confidence Interval (CI).

Supplementary Table 9. Regression coefficients from the random effects of physical, verbal and relational victimisation on psychological distress and life satisfaction.

	Psychological Distress			Life Satisfaction		
**Country Name**	**Physical**	**Verbal**	**Relational**	**Physical**	**Verbal**	**Relational**
Albania	0.128	0.132	0.094	-0.069	-0.085	-0.069
United Arab Emirates	0.170	0.249	0.247	-0.113	-0.166	-0.185
Argentina	0.100	0.180	0.163	-0.120	-0.187	-0.205
Austria	0.090	0.167	0.184	-0.137	-0.199	-0.231
Bulgaria	0.104	0.140	0.142	-0.086	-0.142	-0.115
Bosnia and Herzegovina	0.080	0.151	0.132	-0.074	-0.123	-0.145
Belarus	0.139	0.180	0.186	-0.151	-0.182	-0.190
Brazil	0.102	0.156	0.155	-0.096	-0.153	-0.180
Brunei Darussalam	0.036	0.063	0.120	-0.106	-0.080	-0.147
Switzerland	0.098	0.141	0.149	-0.118	-0.182	-0.197
Chile	0.182	0.205	0.202	-0.146	-0.189	-0.193
Colombia	0.079	0.132	0.128	-0.068	-0.137	-0.126
Costa Rica	0.134	0.169	0.164	-0.104	-0.200	-0.209
Czech Republic	0.085	0.157	0.175	-0.113	-0.178	-0.201
Germany	0.080	0.183	0.190	-0.123	-0.210	-0.212
Dominican Republic	0.137	0.179	0.175	-0.061	-0.078	-0.096
Spain	0.084	0.166	0.147	-0.092	-0.179	-0.169
Estonia	0.112	0.157	0.191	-0.110	-0.173	-0.207
Finland	0.151	0.245	0.260	-0.158	-0.234	-0.255
France	0.157	0.238	0.243	-0.131	-0.214	-0.212
United Kingdom	0.219	0.278	0.307	-0.267	-0.287	-0.331
Georgia	0.214	0.264	0.233	-0.089	-0.110	-0.107
Greece	0.079	0.142	0.135	-0.050	-0.109	-0.116
Hong Kong-China	0.159	0.187	0.196	-0.113	-0.125	-0.170
Croatia	0.112	0.158	0.165	-0.121	-0.179	-0.197

Hungary	0.061	0.145	0.171	-0.055	-0.131	-0.176
Indonesia	0.104	0.119	0.119	-0.058	-0.068	-0.070
Ireland	0.142	0.193	0.200	-0.206	-0.244	-0.274
Iceland	0.257	0.269	0.302	-0.188	-0.245	-0.312
Italy	0.050	0.129	0.132	-0.064	-0.127	-0.161
Jordan	0.124	0.132	0.136	-0.163	-0.171	-0.154
Japan	0.215	0.270	0.276	-0.178	-0.207	-0.227
Kazakhstan	0.092	0.128	0.131	-0.061	-0.086	-0.084
Korea	0.244	0.281	0.341	-0.204	-0.227	-0.315
Lithuania	0.119	0.190	0.179	-0.073	-0.127	-0.118
Luxembourg	0.125	0.204	0.216	-0.154	-0.226	-0.232
Latvia	0.116	0.167	0.189	-0.112	-0.157	-0.175
Morocco	0.062	0.125	0.140	-0.053	-0.117	-0.127
Republic of Moldova	0.180	0.209	0.192	-0.163	-0.188	-0.173
Mexico	0.126	0.201	0.185	-0.100	-0.165	-0.152
Malta	0.107	0.168	0.179	-0.083	-0.160	-0.172
Montenegro	0.174	0.196	0.197	-0.077	-0.135	-0.154
Malaysia	0.102	0.153	0.160	-0.128	-0.160	-0.145
Netherlands	0.159	0.241	0.271	-0.139	-0.193	-0.210
Panama	0.072	0.128	0.130	-0.090	-0.138	-0.094
Peru	0.158	0.236	0.212	-0.121	-0.160	-0.181
Philippines	0.056	0.070	0.077	-0.068	-0.011	-0.056
Poland	0.121	0.201	0.190	-0.104	-0.190	-0.197
Portugal	0.092	0.176	0.183	-0.106	-0.176	-0.199
Qatar	0.175	0.239	0.228	-0.090	-0.145	-0.163
Romania	0.130	0.174	0.142	-0.143	-0.160	-0.139
Russian Federation	0.163	0.232	0.209	-0.076	-0.144	-0.169
Saudi Arabia	0.177	0.236	0.210	-0.073	-0.115	-0.111
Serbia	0.185	0.237	0.228	-0.038	-0.111	-0.113
Slovak Republic	0.074	0.133	0.141	-0.060	-0.097	-0.141
Slovenia	0.120	0.198	0.180	-0.110	-0.177	-0.218

Sweden	0.194	0.283	0.302	-0.164	-0.234	-0.275
Thailand	0.097	0.137	0.129	-0.097	-0.109	-0.116
Turkey	0.143	0.196	0.190	-0.085	-0.142	-0.187
Ukraine	0.143	0.209	0.192	-0.128	-0.183	-0.184
Uruguay	0.154	0.190	0.190	-0.077	-0.132	-0.149
United States	0.202	0.285	0.270	-0.204	-0.274	-0.273
Vietnam	0.170	0.226	0.214	-0.102	-0.144	-0.152

Note: Regression coefficients were taken from Model 3 when analyses by subtypes for psychological distress and life satisfaction were conducted

Supplementary Table 10. Moderating effects of country-level factors on the association between physical bullying victimisation and psychological distress.

	Models		
	**Bullying Prevalence**	**Gini index**	**GDP**
**Physical Bullying Victimisation**	0.194<br>(0.156, 0.233)<br>***p* = 0.000**	0.162<br>(0.095, 0.229)<br>***p* = 2.28e-06**	-0.097<br>(-0.109, -0.085)<br>***p* = 0.000**
**Bullying Prevalence**	0.310<br>(-0.407, 1.0.28)<br>*p* = 3.97e-01		
**Physical Bullying Victimisation* Bullying Prevalence**	-0.229<br>(-0.360, -0.0698<br>***p* = 6.16e-04**		
**Gini index**		0.001<br>(-0.007, 0.010)<br>*p* = 7.45e-01	
**Physical Bullying Victimisation*Gini index**		-0.001<br>(-0.003, 0.001)<br>*p* = 3.22e-01	
**GDP**			0.041<br>(0.011, 0.071)<br>***p* = 7.17e-03**
**Physical Bullying Victimisation *GDP**			-0.015<br>(-0.018, -0.012)<br>***p* = 0.000**

All three multilevel linear regression models were adjusted for gender and parental economic, social cultural status (ESCS). All three country-level factors were included as a main effect and as an interaction term with physical bullying victimisation. Asterisk (*) indicated an interaction term. All models were weighted. In each output, the regression coefficients with estimates, 95% confidence intervals and *p* values are displayed. Bold *p* indicated a significant *p* value. GDP indicated GDP per capita purchasing power parity. The Gini index and GDP for each country included in this analysis were sourced from the World Bank 2018 dataset, retrieved 13th January 2023.

Supplementary Table 11. Moderating effects of country-level factors on the association between verbal bullying victimisation and psychological distress.

	Models<br>Bullying Prevalence	 Gini index	 GDP
**Verbal Bullying Victimisation**	0.276<br>(0.240, 0.312)<br>***p* = 0.000**	0.237<br>(0.169, 0.306)<br>***p* = 9.37e-12**	0.158<br>(0.134, 0.183)<br>***p* = 0.000**
**Bullying Prevalence**	0.229<br>(-0.474, 0.931)<br>*p* = 5.23e-01		
**Verbal Bullying Victimisation* Bullying Prevalence**	-0.322<br>(-0.446, -0.199)<br>***p* = 3.25e-07**		
**Gini index**		0.001<br>(-0.007, 0.009)<br>*p* = 8.42e-01	
**Verbal Bullying Victimisation*Gini index**		-0.001<br>(-0.003, 0.004)<br>*p* = 1.25e-01	
**GDP**			0.028<br>(-0.002, 0.058)<br>*p* = 6.40e-02
**Verbal Bullying Victimisation *GDP**			0.008<br>(0.002, 0.014)<br>***p* = 9.82e-03**

All three multilevel linear regression models were adjusted for gender and parental economic, social cultural status (ESCS). All three country-level factors were included as a main effect and as an interaction term with verbal bullying victimisation. Asterisk (*) indicated an interaction term. All models were weighted. In each output, the regression coefficients with estimates, 95% confidence intervals and *p* values are displayed. Bold *p* indicated a significant *p* value. GDP indicated GDP per capita purchasing power parity. The Gini index and GDP for each country included in this analysis were sourced from the World Bank 2018 dataset, retrieved 13th January 2023.

Supplementary Table 12. Moderating effects of country-level factors on the association between relational bullying victimisation and psychological distress.

		Models	
	**Bullying Prevalence**	**Gini index**	**GDP**
**Relational Bullying Victimisation**	0.280<br>(0.241, 0.318)<br>***p* = 0.000**	0.261<br>(0.188, 0.334)<br>***p* = 2.74e-12**	0.149<br>(0.125, 0.173)<br>***p* = 0.000**
**Bullying Prevalence**	0.183<br>(-0.549, 0.915)<br>*p* = 6.24e-01		
**Relational Bullying Victimisation* Bullying Prevalence**	-0.331<br>(-0.416, -0.201)<br>***p* = 8.18e-07**		
**Gini index**		0.0003<br>(-0.008, 0.009)<br>*p* = 9.43e-01	
**Relational Bullying Victimisation*Gini index**		-0.002<br>(-0.004, 0.0001)<br>***p* = 4.03e-02**	
**GDP**			0.033<br>(0.002, 0.063)<br>***p* = 3.75e-02**
**Relational Bullying Victimisation *GDP**			0.011<br>(0.005, 0.017)<br>***p* = 3.03e-04**

All three multilevel linear regression models were adjusted for gender and parental economic, social cultural status (ESCS). All three country-level factors were included as a main effect and as an interaction term with relational bullying victimisation. Asterisk (*) indicated an interaction term. All models were weighted. In each output, the regression coefficients with estimates, 95% confidence intervals and *p* values are displayed. Bold *p* indicated a significant *p* value. GDP indicated GDP per capita purchasing power parity. The Gini index and GDP for each country included in this analysis were sourced from the World Bank 2018 dataset, retrieved 13th January 2023.

Supplementary Table 13. Moderating effects of country-level factors on the association between physical bullying victimisation and life satisfaction.

	Models		
	**Bullying Prevalence**	**Gini index**	**GDP**
**Physical Bullying Victimisation**	-0.171<br>(-0.208, -0.134)<br>***p* = 0.000**	-0.168<br>(-0.235, -0.102)<br>***p* = 7.09e-07**	0.101<br>(0.089, 0.114)<br>***p* = 0.000**
**Bullying Prevalence**	0.211<br>(-0.430, 0.852)<br>*p* = 5.19e-01		
**Physical Bullying Victimisation* Bullying Prevalence**	0.212<br>(0.087, 0.337)<br>***p* = 8.62e-04**		
**Gini index**		0.003<br>(-0.006, 0.011)<br>*p* = 5.24e-01	
**Physical Bullying Victimisation*Gini index**		0.002<br>(-0.0003, 0.003)<br>*p* = 9.16e-02	
**GDP**			-0.020<br>(-0.044, -0.003)<br>*p* = 9.01e-02
**Physical Bullying Victimisation *GDP**			0.013<br>(0.010, 0.016)<br>***p* = 0.000**

All three multilevel linear regression models were adjusted for gender and parental economic, social cultural status (ESCS). All three country-level factors were included as a main effect and as an interaction term with physical bullying victimisation. Asterisk (*) indicated an interaction term. All models were weighted. In each output, the regression coefficients with estimates, 95% confidence intervals and *p* values are displayed. Bold *p* indicated a significant *p* value. GDP indicated GDP per capita purchasing power parity. The Gini index and GDP for each country included in this analysis were sourced from the World Bank 2018 dataset, retrieved 13th January 2023.

Supplementary Table 14. Moderating effects of country-level factors on the association between verbal bullying victimisation and life satisfaction.

	Models		
	**Bullying Prevalence**	**Gini index**	**GDP**
**Verbal Bullying Victimisation**	-0.265<br>(-0.299, -0.231)<br>***p* = 0.000**	-0.225<br>(-0.297, -0.152)<br>***p* = 1.18e-09**	-0.118<br>(-0.142, -0.095)<br>***p* = 0.000**
**Bullying Prevalence**	0.224<br>(-0.401 0.849)<br>*p* = 4.83e-01		
**Verbal Bullying Victimisation* Bullying Prevalence**	0.377<br>(0.261, 0.493)<br>***p* = 1.62e-10**		
**Gini index**		0.003<br>(-0.005, 0.011)<br>*p* = 4.64e-01	
**Verbal Bullying Victimisation*Gini index**		0.002<br>(-0.003, 0.004)<br>*p* = 8.96e-02	
**GDP**			-0.049<br>(-0.074, -0.025)<br>***p* = 5.95e-05**
**Verbal Bullying Victimisation *GDP**			-0.012<br>(-0.018, -0.006)<br>***p* = 4.46e-05**

All three multilevel linear regression models were adjusted for gender and parental economic, social cultural status (ESCS). All three country-level factors were included as a main effect and as an interaction term with verbal bullying victimisation. Asterisk (*) indicated an interaction term. All models were weighted. In each output, the regression coefficients with estimates, 95% confidence intervals and *p* values are displayed. Bold *p* indicated a significant *p* value. GDP indicated GDP per capita purchasing power parity. The Gini index and GDP for each country included in this analysis were sourced from the World Bank 2018 dataset, retrieved 13th January 2023.

Supplementary Table 15. Moderating effects of country-level factors on the association between relational bullying victimisation and life satisfaction.

	Models		
	**Bullying Prevalence**	**Gini index**	**GDP**
**Relational Bullying Victimisation**	-0.286<br>(-0.326, -0.246)<br>***p* = 0.000**	-0.273<br>(-0.354, -0.191)<br>***p* = 5.17e-11**	-0.123<br>(-0.148, -0.097)<br>***p* = 0.000**
**Bullying Prevalence**	0.320<br>(-0.327, 0.966)<br>*p* = 3.33e-01		
**Relational Bullying Victimisation* Bullying Prevalence**	0.399<br>(0.262, 0.535)<br>***p* = 1.04e-08**		
**Gini index**		0.004<br>(-0.005, 0.012)<br>*p* = 3.75e-01	
**Relational Bullying Victimisation*Gini index**		0.003<br>(0.0005, 0.005)<br>***p* = 1.77e-02**	
**GDP**			-0.054<br>(-0.079, -0.029)<br>***p* = 1.95e-05**
**Relational Bullying Victimisation *GDP**			-0.015<br>(-0.021, -0.009)<br>***p* = 2.64e-06**

All three multilevel linear regression models were adjusted for gender and parental economic, social cultural status (ESCS). All three country-level factors were included as a main effect and as an interaction term with relational bullying victimisation. Asterisk (*) indicated an interaction term. All models were weighted. In each output, the regression coefficients with estimates, 95% confidence intervals and *p* values are displayed. Bold *p* indicated a significant *p* value. GDP indicated GDP per capita purchasing power parity. The Gini index and GDP for each country included in this analysis were sourced from the World Bank 2018 dataset, retrieved 13th January 2023.
